# Comparing retro-cue benefit mechanisms in visual working memory: completely valid vs. highly valid retro-cues

**DOI:** 10.1186/s40359-024-02145-2

**Published:** 2024-11-07

**Authors:** Qiang Liu, Lijing Guo, Dan Nie, Kai Fu, Chaoxiong Ye

**Affiliations:** 1 School of Education, Anyang Normal University, Anyang, 455000 China; 2 Institute of Brain and Psychological Sciences, Sichuan Normal University, Chengdu, 610066 China; 3 Research Center of Brain and Cognitive Neuroscience, Liaoning Normal University, Dalian, 116029 China; 4 Department of Psychology, University of Jyväskylä, Jyväskylä, 40014 Finland; 5 Faculty of Medicine, University of Helsinki, Helsinki, 00014 Finland

**Keywords:** Visual working memory, Retro-cue benefit, Cue validity, Electroencephalography, Attention allocation, Contralateral delay activity, N2-posterior-contralateral

## Abstract

Visual working memory (VWM) plays a crucial role in temporarily maintaining and manipulating visual information. Retro-cue benefit (RCB) refers to the enhancement of memory performance when attention is directed toward a subset of items in VWM after their initial encoding. Our recent electroencephalogram (EEG) studies indicate that cue validity affects the mechanisms underlying RCB formation. However, previous research has not thoroughly examined whether these mechanisms differ between completely valid and highly valid cue conditions. This study investigates the consistency of RCB mechanisms under conditions of complete (100%) and high (80%) retro-cue validity. We manipulated retro-cue validity and examined cognitive processing mechanisms under different validity conditions using EEG. Specifically, we focused on the N2pc component, which reflects attentional resource allocation, and the contralateral delay activity (CDA) component, which reflects the quantity of information retained in VWM. The results, encompassing both behavioral and event-related potential (ERP) findings, show that participants in both the 100% and 80% cue validity conditions exhibit robust RCB. Notably, the degree of RCB remains consistent across these conditions, indicating that participants utilize retro-cues to enhance VWM performance to the same extent. In the 80% cue validity condition, a significant retro-cue cost (RCC) was observed, indicating that participants selectively discarded uncued items from VWM. In invalid trials, response accuracy drops to chance levels, supporting the removal hypothesis. ERP results reveal that attentional resource allocation (N2pc) and the quantity of retained information (CDA) remain uniform across cue validity conditions. The mechanism responsible for RCB formation appears to involve an all-or-nothing process of discarding uncued information rather than a flexible resource allocation strategy. This study provides insights into attention allocation and information-processing mechanisms in VWM, suggesting that conclusions drawn from tasks with completely valid retro-cues can be integrated with findings from highly valid cue tasks. These findings also illuminate the flexibility of internal attentional resource allocation during RCB formation and contribute to our understanding of attention processes in VWM.

## Introduction

 Visual working memory (VWM) plays an essential role in cognitive processing by temporarily storing and manipulating visual information to meet task demands [1, 2]. It is well-known for its constraint of retaining only three to four representations at a time [2–6]. However, VWM can allocate resources flexibly to task-related information while filtering out irrelevant information, thereby compensating for its limited capacity [7–13]. In recent years, a burgeoning body of research has delved into the mechanisms underpinning VWM, revealing its adaptive and dynamic nature, as opposed to a rigid construct [14–26]. This often entails a reallocation of VWM resources toward these specific representations during the maintenance phase [27–40]. Consequently, internal attention mechanisms become imperative in regulating access to VWM and prioritizing extant VWM representations for behavioral output.

The influence of internal attention on VWM has been extensively examined using retro-cues [27]. In a typical retro-cue experiment [28, 30], participants are instructed to retain a memory array for subsequent recall. During the interval between presentation of the memory array and the test array, a retro-cue is presented to indicate the most likely probed item from the memory array. This effect on VWM performance is known as the retro-cue effect (RCE), comprising retro-cue benefit (RCB) and retro-cue cost (RCC). RCB signifies that in the valid retro-cue condition (indicating the item’s location to be tested), memory performance outperforms that of the no-cue or neutral-cue conditions. Conversely, RCC denotes that in the invalid retro-cue condition (pointing to an item that will not be tested), memory performance is worse than that of the no-cue or neutral-cue conditions. Recent studies suggest that RCE occurs not only when retro-cues direct attention toward specific memory items but also when they highlight a particular dimension (e.g., color or orientation) across all items [41–47]. This underscores the complexity of the impact of internal attention on VWM. Thus, investigating the mechanisms behind RCE can deepen our understanding of the cognitive processes involved in attention during VWM maintenance.

Two influential hypotheses have emerged to explain the mechanisms underlying the RCE: the prioritization hypothesis and the removal hypothesis. The prioritization hypothesis suggests that the performance enhancement of a cued item in RCE results from elevating the cued representation to a prioritized state during maintenance without excluding non-cued representations from VWM. The cued representation is enhanced/protected while in the prioritized state, reducing competition with non-cued representations and consequently improving memory performance of the cued item. According to this hypothesis, non-cued representations are maintained continuously in VWM, but are less accessible than cued representations [17, 48, 49]. However, the removal hypothesis posits that the retro-cue serves to reduce memory load by expelling non-cued items from VWM, thereby granting participants more available VWM resources to maintain cued representation. This reduction in inter-representation interference and resource competition is believed to improve memory performance [50–54]. Consequently, the removal hypothesis predicts that retro-cue benefits for cued representation should be accompanied by significant RCC for non-cued representation. Conversely, the prioritization hypothesis predicts that RCC would not be observed theoretically, as the status of the non-cued representations remains unchanged. Therefore, presence or absence of RCC is crucial to discerning between the hypotheses explaining the RCE phenomenon.

RCC has been observed in some studies [28, 38, 55], but other studies using similar retro-cue paradigms have not found such costs [17, 56, 57]. Consequently, while the prioritization and removal hypotheses initially may seem mutually exclusive, they actually may reflect automatic processing strategies that participants employed under different circumstances. The study by Günseli, et al. [58] suggests that whether non-cued representations are removed from VWM could depend on the expectation of retro-cue validity [58]. They discovered that retro-cue benefits were observed consistently regardless of retro-cue validity, but retro-cue costs became prominent when the retro-cue had high validity (i.e., 80% cue validity). Furthermore, retro-cue costs were absent for memory performance when the retro-cue had low validity (i.e., 50% cue validity). These findings suggest that participants strategically implemented the retro-cue during the VWM task. When the cue is relatively unreliable, participants prioritize the cued representation for maintenance without excluding non-cued representations. Conversely, when the cue is highly reliable, participants not only prioritize, but also discard, non-cued representations during maintenance, resulting in notable retro-cue costs when a non-cued item is tested. However, previous studies related to retro-cue benefits often overlooked the impact of cue validity on the mechanisms of retro-cue benefits. While the study by Günseli, et al. [58] has directed attention towards the role of retro-cue validity, due to the inherent limitations of behavioral experiments in providing direct evidence regarding whether individuals retain non-cued representations in VWM, the results from behavioral experiments could not yield sufficiently compelling conclusions.

Given the advantages of direct brain activity observation and the high temporal resolution of electroencephalogram (EEG) technology, researchers have extensively used event-related potential (ERP) measurements to study VWM storage. The EEG technique has been applied extensively in investigating RCB [48, 50–52, 59, 60]. A frequently employed ERP component in RCB is contralateral delay activity (CDA), a sustained negative potential reflecting the information currently held in VWM [61–67]. CDA is characterized by a large negative wave observed over posterior electrode sites contralateral to the locations of the stored objects. It persists throughout the memory retention interval in change detection tasks and is strongly modulated by the number of representations in VWM, reaching an asymptote once capacity is exhausted. This ERP component also has been utilized to investigate the impact of retro-cue validity on RCB mechanisms [68, 69]. For example, in our recent study employing CDA as an index of VWM storage, we manipulated retro-cue validity, examining the fate of non-cued representations in VWM when retro-cue validity was set at 80% (high retro-cue validity) and 20% (low retro-cue validity). The results revealed that although participants shifted their attention based on the cue in both high and low retro-cue validity conditions, they only maintained non-cued representations in the low retro-cue validity condition, but removed non-cued representations from VWM in the high retro-cue validity condition [69]. This study supports the hypothesis proposed by Günseli, et al. [58] and provides more direct evidence than behavioral experiments, suggesting that retro-cue validity may impact the mechanisms underlying RCB.

While our recent research has provided insights into the influence of retro-cue validity on RCB mechanisms [69], a comprehensive understanding of how retro-cue validity impacts RCB mechanisms remains an ongoing pursuit. Notably, previous research on RCB mechanisms has employed retro-cues that consistently were 100% valid, with no consideration of invalid cue conditions. Moreover, many existing hypotheses regarding RCB mechanisms have been proposed under the assumption of 100% retro-cue validity [30, 32, 35, 36, 51]. However, cognitive processes may exhibit qualitative distinctions between performing a retro-cue task with 100% retro-cue validity and one with high retro-cue validity, such as 80% cue validity. In the 80% retro-cue valid task, participants still may have the incentive to retain uncued items during the test phase, as they might be tasked with recalling these uncued items. However, in the 100% retro-cue valid task, participants lack any motivation to retain uncued representations. This motivational divergence could result in differences in RCB mechanisms between the two retro-cue validity conditions. While our previous research found that participants can remove uncued representations to some extent in high retro-cue validity (e.g., 80% cue validity) conditions, it remains uncertain whether this removal process aligns with the mechanisms governing RCB when retro-cues are 100% valid. Only by scrutinizing distinctions in mechanisms between high retro-cue validity tasks and tasks with completely valid retro-cues can we integrate the findings obtained from high retro-cue validity tasks with those from previous tasks involving completely valid retro-cues.

Consequently, the present study employed ERP techniques to investigate retro-cue validity’s influence on RCB mechanisms further. We examined RCB mechanisms in both high retro-cue validity (i.e., 80% cue validity) and completely valid retro-cue (i.e., 100% cue validity) tasks and made comparisons between the similarities and differences in these RCB mechanisms under these two retro-cue validity conditions. In terms of ERP components, we used N2-posterior-contralateral (N2pc) component, which reflects attentional allocation, and the CDA component, which serves as an indicator of VWM storage. The N2pc component has been used widely in extant research to examine deployment of attention and the onset of attentional engagement [70–75]. Both of the N2pc and CDA components have been used in our previous studies that examined the impact of retro-cue validity on RCB mechanisms [69]. In our previous study [69], participants were required to encode and maintain the same number of items in both hemifields simultaneously. If participants continued to maintain all items in VWM, no asymmetry would be apparent in the EEG signal (i.e., no CDA would emerge). However, if non-cued items (particularly from the hemifield opposite the cued item) were dropped from VWM, a CDA would be expected to emerge, indicated by stronger negativity contralateral to the cued item. This approach used CDA to indirectly observe whether non-cued representations were removed from VWM. Since CDA amplitude is thought to track the number of stored items in the visual hemifield within VWM, the current study aimed to use the CDA component more directly. Similar to many traditional VWM studies using CDA [61, 62, 67, 76, 77], we provided participants with a pre-cue arrow before the memory array appeared, instructing them to store only the items in the cued hemifield. This approach allowed the CDA component to directly track the number of items in the cued hemifield stored in VWM. If the VWM representations stored under the completely valid retro-cue condition are more than those stored under the high retro-cue validity condition, we should observe a significantly larger CDA amplitude in the completely valid retro-cue condition compared to the high retro-cue validity condition.

Therefore, in the present study, we hypothesized two potential outcomes. First, there may be significant differences in RCB mechanisms between the high and completely valid retro-cue conditions. While participants in the high retro-cue validity condition can eliminate uncued representations partially, this removal may not be as comprehensive as in the completely valid retro-cue condition. Consequently, participants in the high retro-cue validity condition may retain more VWM information following the retro-cue, resulting in a greater CDA amplitude compared with the completely valid retro-cue condition. Conversely, the second possibility is that no differences exist in RCB mechanisms between the high retro-cue validity condition and the completely valid retro-cue condition. Participants in both retro-cue validity conditions possessed the ability to eliminate uncued representations entirely. In this case, we expected to observe identical CDA components in both retro-cue validity conditions.

## Materials and methods

### Participants

Adequate statistical power for the t-test comparison was ensured by conducting an a priori power analysis. This analysis, performed using G*Power 3.1.9.2 [78], was based on the predicted effect size derived from our previous study [69]. Anticipating a large effect size (Cohen’s d = 0.80) for our experimental design, and setting a statistical power of 80% alongside an alpha level of 0.05, the analysis recommended a total sample size of 15 participants.

All participants in this study volunteered and were university students from Liaoning Normal University between the ages of 18 and 26, with an average age of 23.12 ± 6.12 years (mean ± standard deviation). The sample included 18 participants (six males, 12 females; all right-handed) with normal color vision and either uncorrected or corrected-to-normal vision. Following completion of the experiment, each participant received compensation at a rate of ¥30 per hour. Data from three participants whose behavioral performance was below chance levels were excluded from analysis. Consequently, data from the remaining 15 participants were analyzed for the study. Prior to the experiment, written informed consent was obtained from each participant. All procedures adhered to the Declaration of Helsinki guidelines and were approved by Liaoning Normal University’s ethics committees.

### Experimental materials

The experimental paradigm for the retro-cue task was created using E-Prime 2.0. The memory array comprised eight colored squares, with their positions remaining constant throughout the experiment. These specific positions and sizes of these squares were in line with the study by Kuo, et al. [51]. Eight positions were designated to display the memory array, with four positions in each hemifield. These positions were arranged based on two imaginary concentric circles with radii of approximately 3.06° and 5.44° visual angle. Notably, squares positioned on the smaller circles measured 0.77° in size, while those on the larger circles measured 1.36°. The squares’ colors were selected randomly from a pool of eight highly distinguishable colors: red; yellow; blue; green; magenta; purple; orange; and cyan. Stimuli were presented on a 19-inch CRT monitor, with participants seated 70 cm away from the monitor inside a quiet, noise-free experimental room.

### Experimental design

**Fig. 1:**
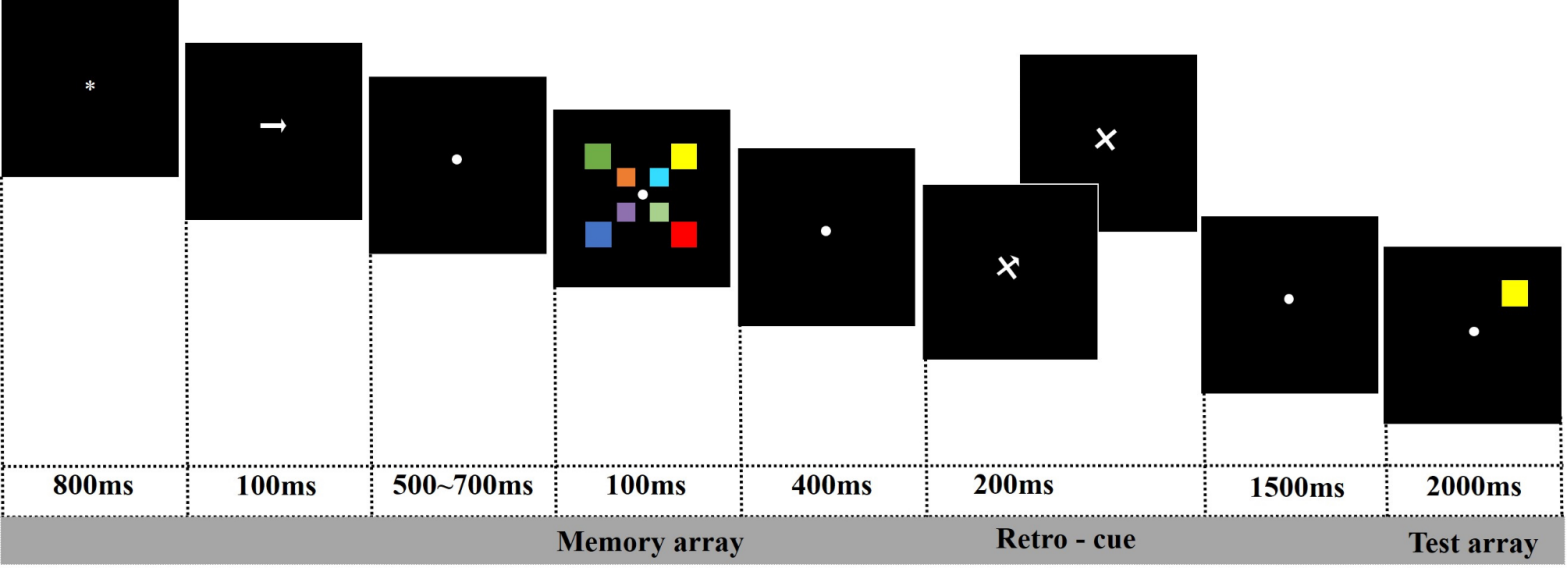
Experimental procedure. The lateralized change detection task involved presenting participants with a memory array comprising four colored squares on each side (100 ms), a retro-cue (200 ms), and a test array (2,000 ms). During the retro-cue trials, a spatial cue (with either 80% or 100% validity) was presented after the memory array. During the neutral trials, a neutral cue without spatially informative orientation was presented after the memory array and interval. Participants were required to determine whether the colors of the squares in the test array in corresponding positions matched those in the memory array

The participants needed to conduct a lateralized change-detection task. The experimental procedure commenced with the presentation of experimental instructions in the center of the screen, then the experimenter explained the study to participants to ensure that they fully comprehended the instructions. The experiment was divided into practice and formal trials. Participants first completed 30 practice trials with retro-cues that were 100% valid. Once participants were familiar with the experimental procedure through practice trials, they initiated formal trials by pressing the “Q” key. As illustrated in Fig. 1, a black background screen initially displayed a fixation point for 800 ms, followed by a left- or right-pointing arrow for 100 ms, indicating which side of the fixation point participants were required to remember the colored squares. The left or right arrow was presented with equal probability and randomized. After a blank screen interval lasting 500–700 ms, a memory array appeared on the screen, comprising four colored squares on each side. However, participants were instructed to remember only the four squares on the side indicated by the preceding arrow cue. The memory array was presented for 100 ms, followed by a 400 ms blank screen interval. Subsequently, a retro-cue was presented for 200 ms, which could be a spatial cue or neutral cue, both presented with equal and random probabilities. The spatial cue (pointing to the upper left, upper right, lower left, or lower right) directed attention toward two of the four squares that needed to be remembered. After the retro-cue disappeared, a 1500 ms blank screen was followed by the probe stimulus. Participants were tasked to determine whether the colors of the squares in corresponding positions matched those in the memory array. The probe array in the cued hemifield had a different color than the memory array on 50% of the trials and was identical in the remaining trials. Participants responded by pressing the “F” key for “same” or the “J” key for “different.” After participants responded, the probe stimulus disappeared, and the next trial began.

The experiment comprised two conditions: one with cues being completely valid (100% cue validity) and the other with cues being highly valid (80% cue validity). The experiment was divided into two blocks based on the cue validity conditions, with each block consisting of 240 trials. The block with completely valid cues contained 120 trials with valid spatial cues and 120 trials with neutral cues, but the block with highly valid cues contained 96 trials with valid spatial cues, 24 trials with invalid spatial cues, and 120 trials with neutral cues. Based on our previous research [39], which suggests that the sequence of experimental blocks can impact participants’ memory strategies and performance outcomes, we took measures to ensure that participants placed complete trust in cue validity in the block with completely valid cues. To achieve this, all participants completed the experiment first in this condition (100% cue validity) before proceeding to the block with highly valid cues (80% cue validity). Prior to commencing the block with highly valid cues, participants were informed explicitly that cue validity in the upcoming experimental block would be reduced to 80%. Furthermore, during the formal experiment, participants were provided with three breaks (one after every 120 trials), each lasting at least 30 s.

### Data analysis

A significance level of *p* < 0.05 was used for all statistical tests. Partial eta squared (ŋ²) was used for effect size in the ANOVAs, and Cohen’s d was used for effect sizes in the t-tests. Bayes factor analyses were used to show whether the t-test results supported the alternative hypothesis or the null hypothesis [79]. The Bayes factor (BF_01_) provides an odds ratio for null/alternative hypotheses (values > 1 favor the null hypothesis, and values < 1 favor the alternative hypothesis). For instance, a BF_01_ of 3 indicates that the null hypothesis is three times more likely than the alternative hypothesis. The results on difference waves were corrected for multiple comparisons using false discovery rate (FDR) correction [80] at a statistical threshold of *p* < 0.05. For statistical significance within the FDR-corrected time windows, fewer than five consecutive time-sampling points were considered nonsignificant, while more than five consecutive time points were deemed significant.

#### Behavioral data analysis

Behavioral data were analyzed, initially involving computation of accuracy and reaction times using E-Prime 2.0 software. A repeated-measures ANOVA with validity blocks (100% cue validity, 80% cue validity) and cue type (valid, neutral) was conducted for accuracy and reaction times. Subsequently, paired-sample t-tests were conducted to evaluate differences between valid and neutral cues in the completely valid cue (100% cue validity) block, as well as between valid, neutral, and invalid cues in the highly valid cue (80% cue validity) block.

#### EEG data preprocessing

EEG data were collected using a 64-electrode cap, following the international 10–20 system, with left and right mastoid references. Electrodes F7 and F8 were positioned approximately 1 cm from the outer canthi of the eyes to monitor horizontal eye movements (HEOG). The EEG signals were digitized at a 24-bit resolution with a sampling rate of 512 Hz and were recorded without online filtering. EEG data analysis was performed using Matlab and Letswave7. Preprocessing of the EEG data involved using a 30 Hz low-pass filter and re-referencing the data to the average of the left and right mastoid electrodes (M1 and M2). Continuous EEG data were segmented into epochs from -600 ms to 1600 ms relative to the retro-cue onset in each trial. Ocular artifacts were removed using Independent Component Analysis (ICA), and threshold artifact rejection was applied to exclude epochs with voltages exceeding ± 100 µV at PO7/PO8 from -500 to 1600 ms (starting from the memory array onset). Additionally, visual inspection was employed to reject any remaining artifacts.

#### Analysis of N2pc and CDA amplitudes

As with some recent N2pc and CDA studies [66], we selected the PO7/PO8 electrodes for analyzing N2pc and CDA amplitudes, using a 100 ms period prior to memory array onset as the baseline (-600 to -500 ms, time-locked to the retro-cue onset). For both N2pc and CDA components, contralateral waveforms were computed as the average of activity recorded at the left hemisphere electrode sites when pre-cue arrows pointed to the right side of the memory array, and the average of activity recorded at the right hemisphere electrode sites when arrows pointed to the left side. Ipsilateral waveforms were computed by averaging the left or right hemisphere sites when pre-cue arrows pointed to the left or right side of the memory array, respectively. The difference waveforms were defined by subtracting ipsilateral activity from contralateral activity.

Given that other memory and cognitive processes were likely present before the retro-cue appeared, we used memory array onset with baseline correction (100 ms before the memory array onset) during EEG analysis, as done in previous studies [50, 60]. We focused on the VWM maintenance stage after retro-cue onset. Considering the key research question was to examine cognitive processing differences in the valid cue conditions of blocks with 100% cue validity versus 80% cue validity, we concentrated our analysis on the N2pc and CDA components in the valid cue conditions of these two cue validity blocks.

**Fig. 2:**
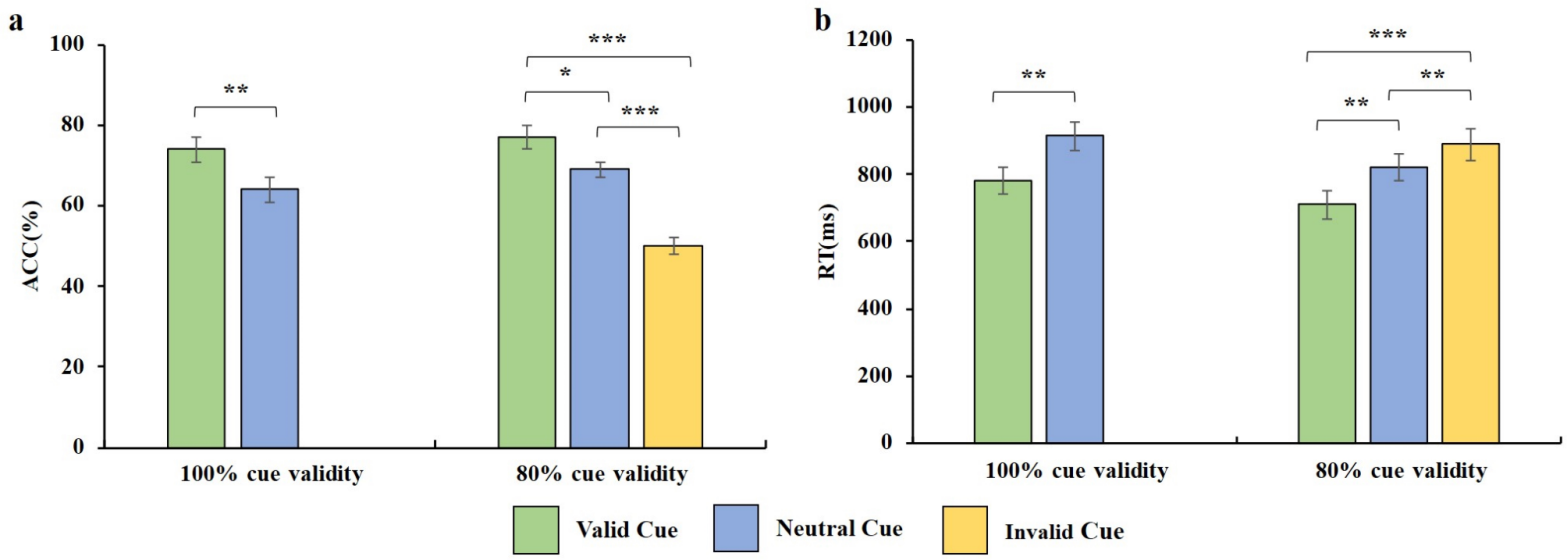
Behavioral results on accuracy (ACC) and reaction time (RT) in each condition. **(a)** Mean ACC and standard error in 100% and 80% cue validity conditions were separated based on cue type factors. **(b)** Mean RT and standard error in 100% and 80% cue validity conditions were separated based on cue type factors. The error bars indicate the standard error of mean. * = *p* < 0.05, ** = *p* < 0.01, *** = *p* < 0.001

Previous studies have shown that N2pc typically occurs 170–220 ms after retro-cue onset, and CDA occurs from 300 ms post-cue and persists throughout maintenance [62, 63, 68, 69, 76, 81–83]. Accordingly, our time window of interest for N2pc was between 170 ms and 220 ms after retro-cue onset, and for CDA, it was between 300 ms and 1600 ms after retro-cue onset. The amplitudes of the difference waves (contralateral minus ipsilateral) at each time point across the entire time window were calculated under both 100% and 80% cue validity blocks. For within-condition testing, we conducted a one-tailed one-sample t-test against zero [84] with FDR correction [80] at each time point to test for the presence of a significant lateralized component. The mean amplitudes of N2pc and CDA across the time window of interest (170–220 ms for N2pc; 300–1600 ms for CDA) under 100% and 80% cue validity conditions were compared to zero using a one-sample t-test (as indicated by the green line on the difference wave in Fig. 2c and d).

Notably, distinct N2pc and CDA components were observed within specific time windows. The sharp negative peak around 200 ms after retro-cue onset was identified as N2pc, while the slow negative wave from 400 ms to 800 ms was identified as CDA. Based on the defined criteria for these two metrics, the time windows of interest were selected as 170–220 ms post retro-cue onset (N2pc) and 300–1000 ms post retro-cue onset (CDA). Average amplitudes of the difference waves for valid cue conditions in both cue validity blocks within these time windows were computed, and paired-sample t-tests were conducted to compare these component amplitudes (N2pc and CDA) between the 100% cue validity and 80% cue validity blocks.

## Results

### Behavioral results

**Fig. 3:**
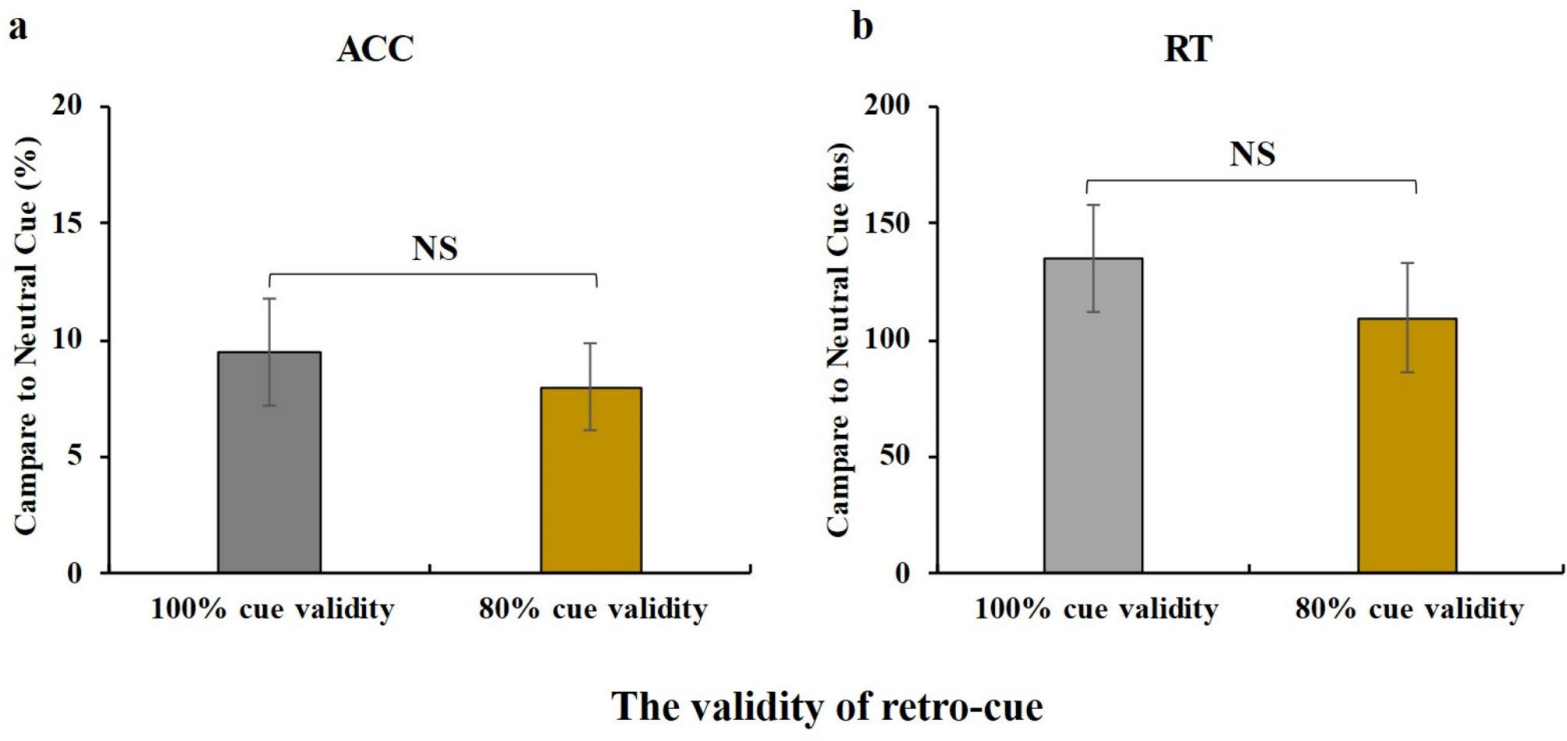
Retro-cue benefit (RCB) under different cue validity conditions. **(a)** RCB on mean accuracy (ACC) under the 100% and 80% cue validity conditions. **(b)** RCB on mean reaction time (RT) under 100% and 80% cue validity conditions. Error bars indicate the standard error of mean. NS = non-significant

The behavioral results are presented in Fig. 3. For accuracy, a significant main effect of validity condition on accuracy was observed (F(1,14) = 15.871, *p* = 0.001, ŋ² = 0.959). Similarly, a significant main effect of cue type on accuracy was found (F(1,14) = 22.113, *p* < 0.001, ŋ² = 0.992). However, the interaction between condition and cue type on accuracy was not significant (F(1,14) = 0.338, *p* = 0.570, ŋ² = 0.084). Follow-up comparisons revealed that under the 100% cue validity condition, accuracy for valid cue trials was significantly higher than that for neutral cue trials (valid cue: 0.74 ± 0.12; neutral cue: 0.64 ± 0.12; t(14) = 3.812, *p* = 0.002, BF_01_ = 0.045, Cohen’s d = 0.83). Similarly, under the 80% cue validity condition, accuracy for valid cue trials was significantly greater than for neutral cue trials (valid cue: 0.77 ± 0.12; neutral cue: 0.69 ± 0.08; t(14) = 4.13, *p* = 0.01, BF_01_ = 0.026, Cohen’s d = 0.78), while accuracy for neutral cue trials was higher than for invalid cue trials (invalid cue: 0.50 ± 0.08; t(14) = 5.59, *p* < 0.001, BF_01_ = 0.002, Cohen’s d = 2.65).

As for reaction time, a significant main effect of validity condition on reaction time was found (F(1,14) = 19.484, *p* = 0.001, ŋ² = 0.984), as well as a significant main effect of cue type (F(1,14) = 28.781, *p* < 0.001, ŋ² = 0.999). However, the interaction between condition and cue type on reaction time did not reach statistical significance (F(1,14) = 2.659, *p* = 0.125, ŋ² = 0.33). Follow-up comparisons showed that under the 100% cue validity condition, reaction times for valid cue trials were significantly shorter than for neutral cue trials (valid cue: 779.7 ± 152.98; neutral cue: 914.6 ± 161.12; t(14) = 3.812, *p* = 0.002, BF_01_ = 0.045, Cohen’s d = 0.86). Similarly, under the 80% cue validity condition, reaction times for valid cue trials were significantly shorter than those for neutral cue trials (valid cue: 710 ± 163.44; neutral cue: 819.6 ± 157.24; t(14) = 4.487, *p* = 0.001, BF_01_ = 0.014, Cohen’s d = 0.68), whereas reaction times for neutral cue trials were shorter than those for invalid cue trials (invalid cue: 889.2 ± 187.07; t(14) = 3.549, *p* = 0.003, BF_01_ = 0.071, Cohen’s d = 0.40). Furthermore, a significant difference in reaction times between valid and invalid cue trials was found (t(14) = 6.606, *p* < 0.001, BF_01_ < 0.001, Cohen’s d = 1.02).

**Fig. 4:**
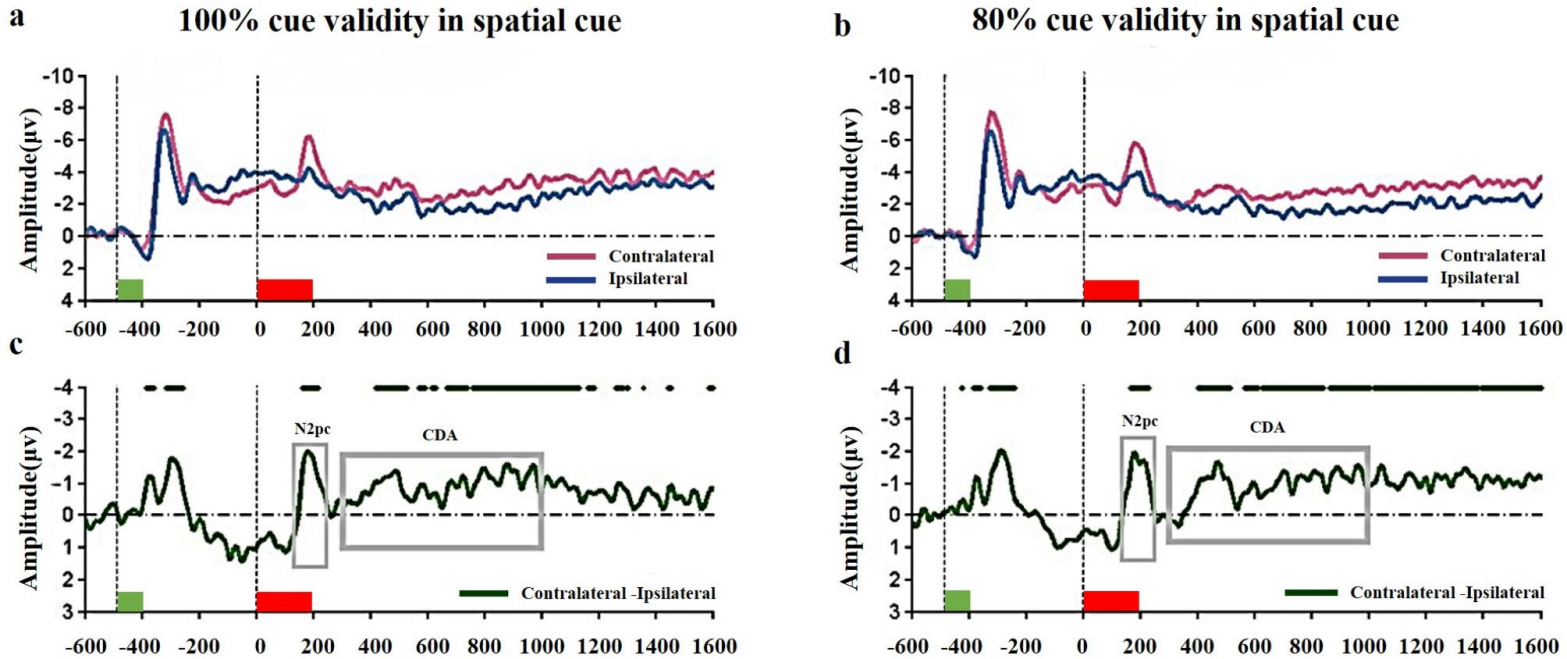
Grand average ERPs and difference waves time-locked to the onset of the retro-cue array. **(a)** Grand average ERPs for the valid cue condition under the 100% cue validity condition. The green and red rectangles on the x-axis show the timing of the memory array (-500 – -400 ms) and retro-cue (0–200 ms). Pink lines represent activity contralateral to, and blue lines represent activity ipsilateral to, the lateralized memory stimuli. **(b)** Grand average ERPs for the valid cue condition under the 80% cue validity block. **(c)** Mean ERP difference wave form for the valid cue condition under the 100% cue validity block. The black lines above the waveforms indicate amplitudes significantly larger than zero throughout the entire duration. The gray-boxed areas denote the analysis time window used to compute the mean N2pc (170–220 ms) and CDA amplitude (300–1000 ms). **(d)** Mean ERP difference wave form for the valid cue condition under the 80% cue validity block. The gray-box areas denote the analysis time window used to compute the mean N2pc and CDA amplitude

Importantly, under the 100% cue validity condition, the extent of retro-cue benefit in accuracy (100% cue validity: 0.10 ± 0.09; 80% cue validity: 0.08 ± 0.07; t(14) = 0.5814, *p* = 0.570, BF_01_ = 4.381, Cohen’s d = 0.25) and reaction time (100% cue validity: 134.9 ± 90.01; 80% cue validity: 109.6 ± 98.03; t(14) = 1.631, *p* = 0.125, BF_01_ = 1.600, Cohen’s d = 0.29) did not significantly differ from that observed under the 80% cue validity condition (see Fig. 4).

### EEG results

The EEG findings are presented in Fig. 2. The upper segment displays the average waveforms at electrodes PO7 and PO8 for contralateral and ipsilateral responses under the 100% and 80% cue validity conditions with spatial cues. Contralateral and ipsilateral references are with respect to the visual field containing the array of colored squares to be memorized. The lower segment displays the difference waves obtained by subtracting contralateral from ipsilateral responses for spatial cues in both conditions.

**Fig. 5:**
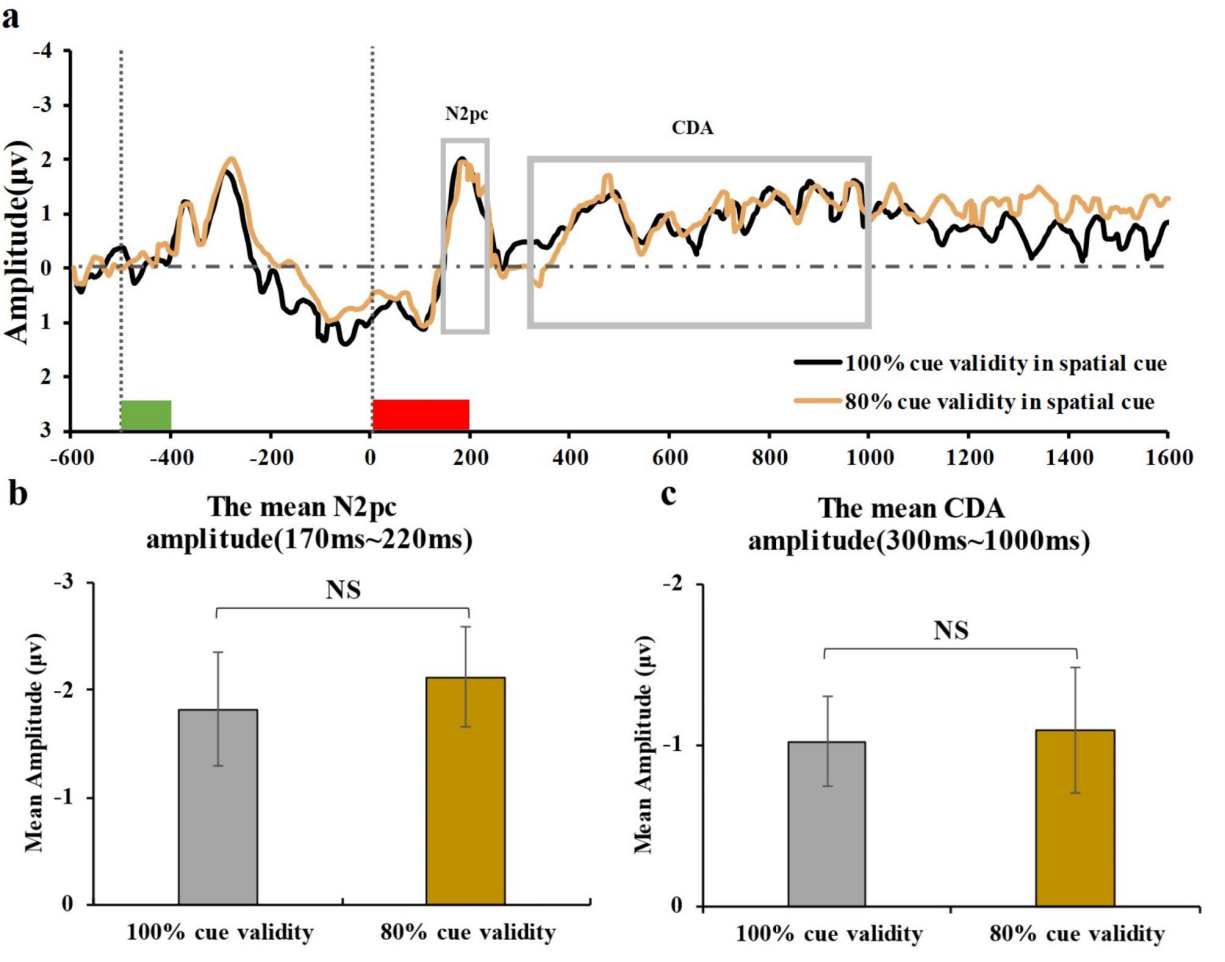
Difference waves during the entire time window and the ERP results. **(a)** Difference wave forms (contralateral waves minus ipsilateral waves) of average ERPs are depicted under different cue validity. The green and red rectangles on the x-axis show the timing of the memory array (-500 – -400 ms) and retro-cue (0–200 ms). The gray-box areas indicate the analysis time window used to calculate the mean N2pc amplitude (170ms ∼ 220ms) and mean CDA amplitude (300ms ∼ 1,000ms). **(b)** Mean N2pc amplitude and standard error for valid cue trials in 100% and 80% cue validity conditions. **(c)** Mean CDA amplitude and standard error for valid cue trials in 100% and 80% cue validity conditions. Error bars indicate the standard error of mean. NS = non-significant

The comparison of average wave amplitude within the windows of interest is presented in Fig. 5. The comparison showed no significant differences in N2pc average wave amplitude between the 100% and 80% cue validity conditions (100% cue validity: -1.82 ± 2.06; 80% cue validity: -2.12 ± 1.82; t(14) = 0.902, *p* = 0.383, BF_01_ = 3.519, Cohen’s d = 0.15). Furthermore, no significant differences were found in average CDA wave amplitude between the 100% and 80% cue validity blocks (100% cue validity: -1.02 ± 1.08; 80% cue validity: -1.09 ± 1.51; t(14) = 0.161, *p* = 0.874, BF_01_ = 5.081, Cohen’s d = 0.05). These results showed no significant differences in the average amplitude of difference waves between the two cue validity blocks within the two time windows of interest.

## Discussion

This study investigated whether the mechanisms underlying RCB formation are consistent across completely valid (100%) and highly valid (80%) retro-cue conditions. Our behavioral and ERP findings revealed that participants exhibited a reliable RCB effect under both 100% and 80% cue validity conditions. Notably, the mechanisms involved in RCB generation, allocating attentional resources and the processing of stored information, exhibited no discernible differences across these two levels of retro-cue validity.

Our behavioral results show that robust RCB is present in both 100% and 80% cue validity conditions. Importantly, the extent of RCB is entirely equivalent across these two cue validity conditions, signifying that participants in both conditions use the retro-cue to enhance their memory performance to the same degree. Moreover, under the 80% cue validity condition, performance in the invalid cue trials was significantly worse than in the neutral cue trials, indicating a substantial RCC. This indicates that participants selectively discarded uncued items from their VWM. Notably, in the invalid trials of the 80% cue validity condition, participants’ response accuracy plummets to chance levels. These behavioral outcomes collectively suggest that in the context of the 80% cue validity condition, participants fully discard uncued items in their VWM. This observation aligns with prior behavioral studies supporting the removal hypothesis [50–54], and also is in line with findings from our previous EEG study [69].

Turning to our EEG results, we observed significant N2pc components under both the 80% cue validity and 100% cue validity conditions. The N2pc results indicate that participants consistently shifted visual attention to the cued location after the retro-cue, irrespective of cue validity. Notably, the patterns and magnitude of attentional resource allocation were similar across both cue validity conditions. Furthermore, our CDA results demonstrate that the quantity of VWM information retained following cue utilization is identical between these two cue validity conditions. This outcome aligns seamlessly with our expectation that no differences exist in RCB mechanisms between the high and completely valid retro-cue conditions. These findings emphasize that the fundamental mechanisms underpinning RCB emergence remain consistent for participants in both the completely valid and high retro-cue validity conditions.

By combining our behavioral and EEG findings, we observed an interesting phenomenon: once retro-cue validity reaches a certain threshold, participants utilize a complete removal mechanism, discarding uncued representations from their VWM to achieve a stable RCB. Notably, the degree of RCB achievement remains consistent as cue validity increases from 80 to 100%. Furthermore, the decrease in cue validity from 100 to 80% does not result in a significant decrease in the additional allocation of attentional resources to the cued region. Future studies should explore whether the removal of uncued information during RCB formation follows an all-or-none process or a graded continuum of resource allocation based on cue validity. It is important to note that our current findings should not be interpreted as evidence that individuals are incapable of flexibly and continuously allocating attention and VWM resources according to task demands. In a previous study [85], researchers used feature-based pre-cues or simultaneous cues to examine the effect of cue validity on CDA and N2pc activity. They similarly found no significant difference between 100% and 75% cue validity conditions. However, further analysis revealed a linear relationship between CDA/N2pc amplitude and cue validity. This suggests that the non-significant difference could still represent a pattern of prioritization with a flexible continuous resource. Thus, future research could examine the flexibility of internal attentional resource allocation during RCB formation by incorporating a wider range of cue validity conditions.

Notably, the experimental paradigm used in this study diverges from our previous investigations into cue validity effects. In our previous study [69], participants were tasked with memorizing information presented bilaterally across the visual field. Conversely, in the present study, participants were instructed only to retain information from one side of the visual field based on the initial arrow cue. This experimental arrangement aligns with the paradigm utilized in previous research examining the RCB effect through the CDA component [51]. The rationale for adopting a unilateral memory paradigm in this study, rather than persisting with our previous paradigm probing cue validity within the bilateral visual field, primarily was to investigate whether participants in the high cue validity condition also would completely forget uncued representations within a unilateral memory context. This prospect emerged from earlier research demonstrating superior performance in VWM when visual items are allocated across both left and right visual fields, predominantly due to participants’ more efficient allocation of attentional resources [86–88]. However, our study ascertained that even in the context of unilateral visual presentation, participants in the high cue validity condition, akin to our previous findings within the bilateral visual field context, could discard uncued representations entirely [69]. This underscores that the mechanism for discarding uncued representations through cues remains unaffected by whether memory stimuli are presented unilaterally or bilaterally.

Our finding that no discernible differences exist in the mechanisms underlying RCB formation between the high and completely valid retro-cue conditions has important implications. It enables us to extend many of the conclusions drawn from tasks with a completely valid retro-cue to findings in tasks involving a highly valid retro-cue, essentially harmonizing these two bodies of research. For instance, in previous research that employed a completely valid retro-cue, it was concluded that the emergence of object-based RCB does not necessitate sustained attention [89, 90]. This conclusion integrates seamlessly with findings from tasks with highly valid cues, suggesting that individuals discard uncued representations from VWM when using highly valid retro-cues, without the need for sustained attention. Consequently, this study establishes a bridge for the smooth amalgamation of conclusions derived from diverse cue validity tasks.

## Conclusions

This study demonstrates that the mechanisms underlying RCB formation are remarkably consistent across both high and completely valid retro-cue conditions. These findings suggest that conclusions from completely valid retro-cue tasks can be effectively integrated with those from highly valid cue tasks. Specifically, individuals tend to employ a complete removal mechanism, effectively discarding uncued representations from their VWM when retro-cue validity reaches a certain threshold. Importantly, the degree of RCB remains consistent as cue validity decreases from 100 to 80%. Furthermore, cue validity augmentation from 100 to 80% does not result in a discernible increase in the additional allocation of resources to the cued region. This suggests that RCB formation follows an all-or-nothing process of discarding uncued information, rather than a flexible, graded allocation of resources depending on cue validity. Overall, these findings offer valuable insights into attentional allocation and information-processing mechanisms in VWM, advancing our understanding of how retro-cue validity influences cognitive processes.

## Data Availability

No datasets were generated or analysed during the current study.

## Abbreviations

CDA: Contralateral delay activity
EEG: Electroencephalogram
ERP: Event-related potential
ICA: Independent component analysis
N2pc: N2-posterior-contralateral
RCB: Retro-cue benefit
RCC: Retro-cue cost
RCE: Retro-cue effect
VWM: Visual working memory
